# PB1-F2 amyloid-like fibers correlate with proinflammatory signaling and respiratory distress in influenza-infected mice

**DOI:** 10.1016/j.jbc.2021.100885

**Published:** 2021-06-17

**Authors:** Christophe Chevalier, Olivier Leymarie, Laura Sedano, Bruno Da Costa, Charles-Adrien Richard, Pauline Maisonnasse, Matthieu Réfregiers, Frédéric Jamme, Ronan Le Goffic

**Affiliations:** 1 Université Paris-Saclay, UVSQ, INRAE, VIM, Jouy-en-Josas, France; 2 Synchrotron SOLEIL, L’Orme des Merisiers, Saint-Aubin, Gif-sur-Yvette, France

**Keywords:** PB1-F2, influenza virus, virulence factor, lung injury, amyloid, inflammation, BAL, bronchoalveolar lavage, BSA, bovine serum albumin, CXCL1, C-X-C motif chemokine ligand 1, IAV, influenza A virus, IL-1β, interleukin-1β, NLRP3, NLR family pyrin domain containing 3, p.i., postinfection, PC, principal component, ROI, region of interest, sDUV, synchrotron-coupled deep UV, sFT-IR, synchrotron-coupled Fourier-transform IR, Trp, tryptophan

## Abstract

PB1-F2 is a virulence factor of influenza A virus known to increase viral pathogenicity in mammalian hosts. PB1-F2 is an intrinsically disordered protein displaying a propensity to form amyloid-like fibers. However, the correlation between PB1-F2 structures and the resulting inflammatory response is unknown. Here, we used synchrotron-coupled Fourier transform-IR and deep UV microscopies to determine the presence of PB1-F2 fibers in influenza A virus–infected mice. In order to study the correlation between PB1-F2 structure and the inflammatory response, transgenic mice expressing luciferase under the control of an NF-κB promotor, allowing *in vivo* monitoring of inflammation, were intranasally instilled with monomeric, fibrillated, or truncated forms of recombinant PB1-F2. Our intravital NF-κB imaging, supported by cytokine quantification, clearly shows the proinflammatory effect of PB1-F2 fibers compared with N-terminal region of PB1-F2 unable to fibrillate. It is noteworthy that instillation of monomeric PB1-F2 of H5N1 virus induced a stronger inflammatory response when compared with prefibrillated PB1-F2 of H1N1 virus, suggesting mechanisms of virulence depending on PB1-F2 sequence. Finally, using whole-body plethysmography to measure volume changes in the lungs, we quantified the effects of the different forms of PB1-F2 on respiratory parameters. Thus, we conclude that PB1-F2–induced inflammation and respiratory distress are tightly correlated with sequence polymorphism and oligomerization status of the protein.

Influenza A virus (IAV), the causative agent of flu, is a major source of respiratory disease worldwide. IAVs are members of the Orthomyxoviridae family, and their genome is constituted of eight segments of single-stranded negative-sense RNA encoding up to 17 proteins (1). According to the World Health Organization, the impact of this pathogen on the human population is estimated to result in about 3 to 5 million cases of severe illness and about 250,000 to 500,000 deaths worldwide each year (http://www.who.int/influenza/en/). IAV spreads through aerosol or by direct contact with respiratory secretions, and it causes acute respiratory tract infections by targeting and replicating within epithelial cells of both upper and lower respiratory tracts. IAV can affect all populations by inducing primary viral pneumonia, a pulmonary manifestation recognized as extremely severe (2). However, many IAV-related complications are likely to develop even in the case of moderate viral pathology, including bacterial superinfection. Indeed, IAV infection predisposes to secondary bacterial infections (secondary pneumonia) by inducing changes in the lung's physical and immunological defenses (3). The most common coinfecting bacterial pathogens are *Streptococcus pneumoniae*, *Staphylococcus aureus*, and *Streptococcus pyogenes* (4). The IAV-associated morbidity is mediated by several virulence factors. Among them, PB1-F2 has been well described for its propensity to promote and increase the severity of secondary bacterial infection (5). PB1-F2 is a small accessory protein encoded by a +1 alternate reading frame of IAV segment 2. This viral protein presents a wide degree of sequence and length polymorphism depending on the viral strain and can be considered as a virulence factor since most of the IAV strains expressing a full-length PB1-F2 are more pathogenic than strains expressing a C-terminally truncated version of the protein. Likewise, in mammalian hosts, laboratory engineered PB1-F2 knockout viruses show a loss of virulence when compared with their WT PB1-F2–expressing counterparts (6, 7, 8). On the contrary, PB1-F2 seems to attenuate pathogenesis in avian hosts (9, 10); a feature in accordance with the hypothesis that the loss of expression of PB1-F2 in mammals is beneficial for viral fitness, whereas in avian species, PB1-F2 is positively selected to contribute to an optimized spreading of the virus without increased virulence (10).

The molecular mechanisms associated with PB1-F2 are still enigmatic, although it is known that its expression during IAV infection is able to promote lung inflammation by enhancing expression of proinflammatory mediators (6, 7, 8, 11). Interestingly, at the molecular level, PB1-F2 is an intrinsically disordered protein, nonstructured in aqueous solution, and capable to switch from a random state to an α-helical or a β-sheet secondary structure depending on the hydrophobicity of its environment (12, 13). Moreover, PB1-F2 has a strong propensity to form cytotoxic soluble oligomers (14) or to fibrillate in amyloid-like fibers in IAV-infected cells depending on cell type (15, 16) and on the form of the expressed protein (*i.e.*, its length). The supramolecular organization of PB1-F2 has been described to be implicated in the alteration of host-cell integrity (17): PB1-F2 amyloid-like fibers display no cytotoxicity when added to mammalian cells, whereas soluble oligomers are highly cytotoxic and disrupt membrane at nanomolar concentrations depending on membrane composition (18). PB1-F2 is initially produced within the infected cell but, because of its membrane disruption activity, PB1-F2 can also be found in the extracellular medium after lysis of the host cell (17). It is thus possible to assay the presence of PB1-F2 in extracellular fluids such as bronchoalveolar lavages from washings of IAV-infected mice (15). Interestingly, a report demonstrated the propensity of the aggregated form of PB1-F2 to activate the NLR family pyrin domain containing 3 (NLRP3) inflammasome pathway extracellularly (19). In contrast, the accumulation of soluble oligomers at the mitochondrial membrane suppresses retinoic acid-inducible gene I protein and NLRP3 signaling pathways (20). In fact, the soluble form of PB1-F2 is able to interact with the Calcoco2 protein leading to modulation of the nature of the innate immune response (21). Therefore, PB1-F2 molecular structure and virulence seem to be correlated. However, the putative role of PB1-F2 amyloid aggregates in the pathogenicity of IAV remains unclear, and new techniques are required to study them properly in the viral context.

In the present work, we harnessed synchrotron UV and IR beamlines to characterize PB1-F2 structures directly within the lungs of infected mice. Using synchrotron-coupled deep UV (sDUV) fluorescence microscopy and taking advantage of the high content of tryptophan (Trp) residues in the PB1-F2 sequence (5/90 amino acids), we correlated the increase of the autofluorescent signal recorded in IAV-infected cells with the synchrotron-coupled Fourier-transform IR (sFT-IR) detection of β-aggregates, as previously demonstrated at the single-cell level (22). Mice were infected with a WT IAV and its PB1-F2 knockout mutant (ΔF2) and were monitored at different times postinfection (p.i.). IR spectra were recorded in the regions of interest (ROIs) identified using sDUV microscopy and subjected to multivariate analysis revealing the presence of β-aggregated structures in mice infected with PB1-F2–expressing IAV. Finally, by delivering recombinant PB1-F2 protein within the lungs of mice, we characterized the PB1-F2 proinflammatory properties and the epithelial damages it provoked in a virus-free context. These damages profoundly alter the respiratory capacities of infected hosts and therefore emphasize the role of PB1-F2 as an important virulence factor of IAVs. Herein, we demonstrate for the first time the tight correlation between the supramolecular structure of PB1-F2 and its associated pathogenicity in mammalian hosts.

## Results

### Mapping of PB1-F2 within the lung architecture

**Figure 1:**
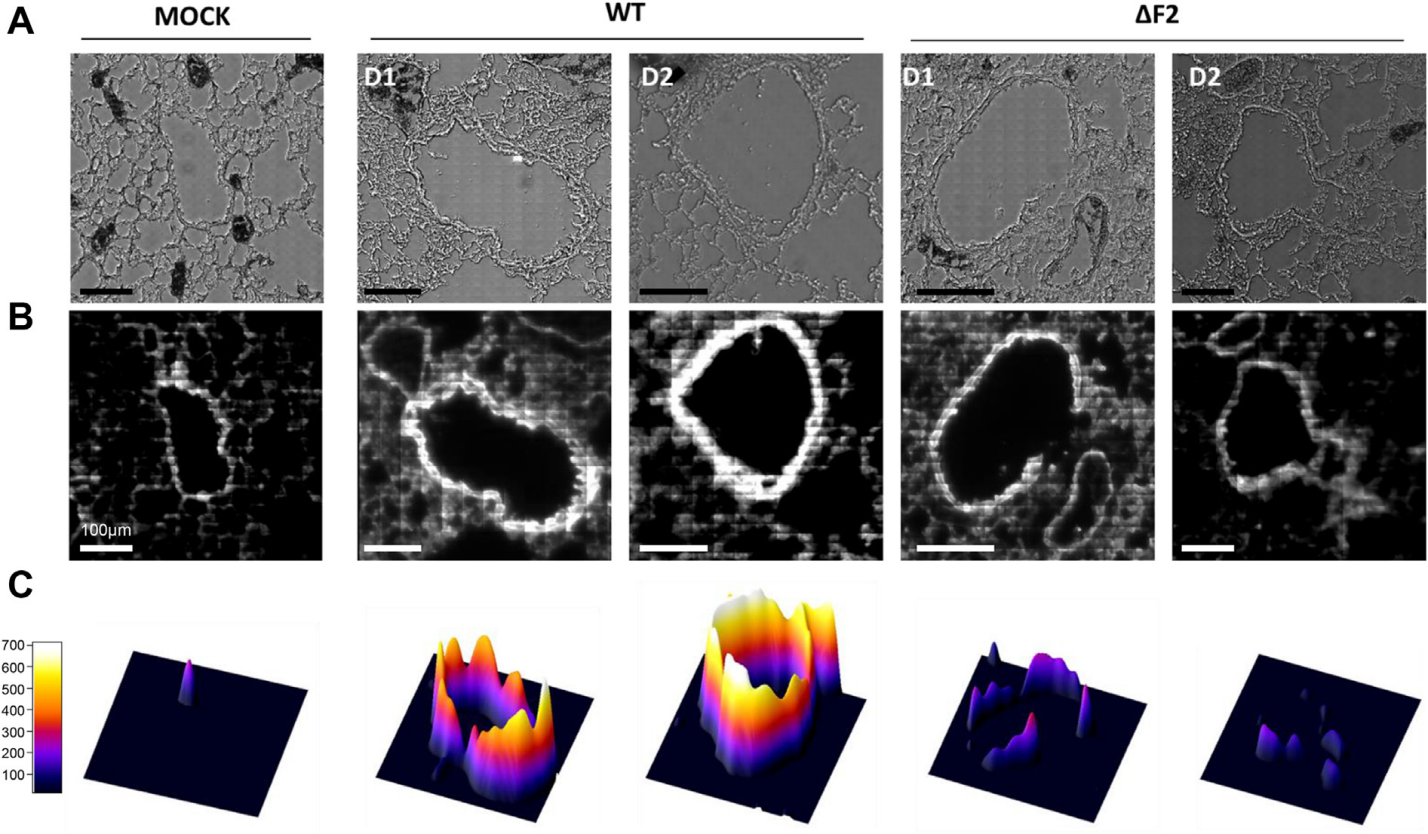
**Synchrotron deep UV (DUV) microspectroscopy comparison of fluorescence intensity between mock-, WT-, and ΔF2-infected lung slices at day 1 and 2 p.i. within the 300 to 380 nm emission range.** For each condition (WT-, ΔF2-, and mock-infected mice), the transmission image in bright field microscopy (*A*) and fluorescent image obtained by DUV fluorescence microscope within the 300 to 380 nm emission range (*B*) are presented. Lung slices were observed with a 100× objective. The scale bars represent 100 μm. *C*, comparison of the fluorescence intensity measured in mock-, WT-, and ΔF2-infected cells. ImageJ software (50) was used to quantify the Trp fluorescent intensity of each region of interest (ROI) and calculate the mean fluorescence normalized to the surface area of the ROI. p.i., postinfection; Trp, tryptophan.

PB1-F2 is known to increase pathogenesis of IAV through exacerbation of the inflammatory processes induced by the infection (5, 6, 11, 19, 23). We hypothesized that because of its propensity to oligomerize and damage cellular membranes, PB1-F2 could induce cytotoxicity and locally impair tissue structure integrity leading to an exacerbation of inflammation. We have previously monitored the impact of PB1-F2 upon infection using sFT-IR and sDUV fluorescence microscopies at the single-cell level (22). Taking advantage of the high content of Trp amino acid in the PB1-F2 sequence (5/90, *i.e.*, 5.5% of the residues), we have previously shown that the increase of the Trp autofluorescence signal is correlated with the presence of PB1-F2 β-aggregated structures in a cell-type–dependent and time-dependent manner. Given that Trp frequency is estimated to be 1.3% in vertebrate proteins (24), we decided to apply this method in order to finely map PB1-F2 aggregated structures in the lungs of IAV-infected mice. To this end, we compared mock-infected mice with mice infected with 1 × 10^6^ PFU of WT A/WSN/1933 (H1N1) virus or with its mutant unable to express PB1-F2 (ΔF2). Mice were euthanized at days 1, 2, and 3 p.i., and lungs were embedded with paraformaldehyde and cut into 8 μm-thick cryosections. sDUV fluorescence of the three sample types was then compared at day 1 and 2 p.i. (Fig. 1). The Trp fluorescence emission intensity was quantified and normalized to the studied area using the 3D surface plot plugin (ImageJ; https://imagej.nih.gov/ij/disclaimer.html). When compared with mock-infected mice, a clear increase of fluorescence was observed into WT IAV-infected mice cryosections and localized within the vicinity of bronchioles, which are known to be a major site of IAV infection (6). This increase of Trp autofluorescence was measured to a lesser extent in ΔF2-infected mice. Thus, sDUV microscopy allowed us to scan the whole surface of the slice of IAV-infected lungs and determine the ROI submitted subsequently to FT-IR microspectroscopic analysis in an attempt to detect the presence of β-aggregated structures. Hence, PB1-F2 appeared to be expressed within bronchioles, and β-aggregated structures are detected at early steps of infection. This feature is known to influence the outcome of the infection (8), suggesting that PB1-F2 could alter the tracheobronchial functions at the beginning of infection.

### PB1-F2 expression is associated with β-aggregated structures within infected bronchioles

**Figure 2:**
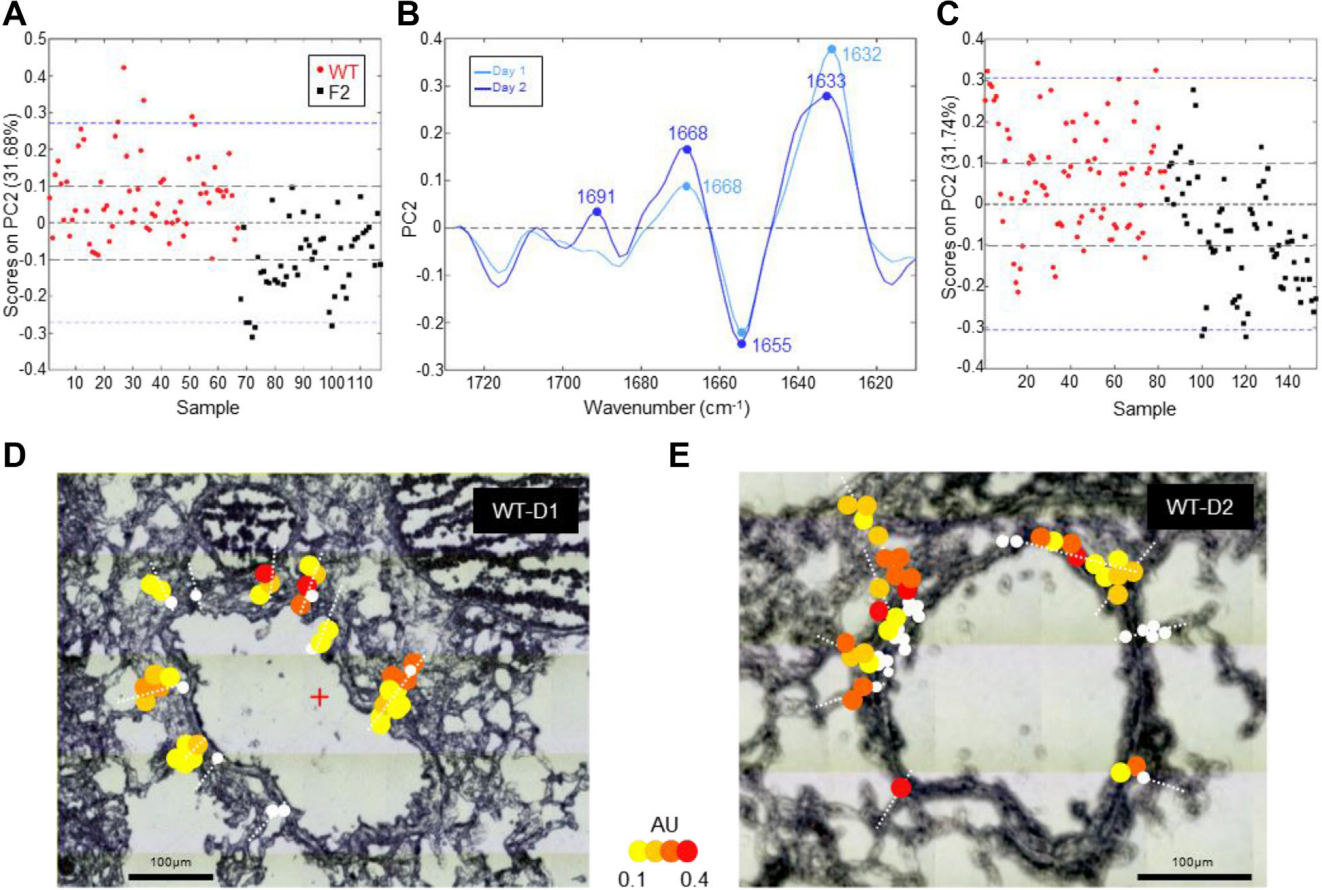
**Synchrotron FT-IR microspectroscopic cartography of secondary structures content of WT and ΔF2-infected IAV-infected lung slices at day 1 and day 2 p.i. within the amide I region.** The second-derivative IR spectra were analyzed by applying PCA. *A* and *C*, score plots of principal component (PC) analysis from the 1700 to 1600 cm^−1^ band IR spectra. The explained variance for PC2 is 31.68% at day 1 (*A*) and 31.74% at day 2 (*C*). *Red circles* and *black squares* correspond to single IR spectra recorded in lung slices of mice infected with virus expressing PB1-F2 (WT) or with PB1-F2 knockout virus (F2), respectively. *B*, loading plot linking the variable space and PC subspace (PC2). At day 1 (*light blue*), PCA score plot shows that the WT and F2 groups are separated along PC2. Compared with F2 group, WT group shows higher IR intensity at 1632 cm^−1^ bands associated with the presence of β-sheet and at 1668 cm^−1^ assigned to β-turn structures (and disordered structures). At day 2, the same tendency is observed: WT and F2 groups are separated along PC2 and characterized by the two peaks present at day 1: 1633 and 1668 cm^−1^. However, it is noteworthy that a third peak at 1691 cm^−1^ appeared in the loading plot. The presence is of great importance since the higher IR intensity at 1633 to 1691 cm^−1^ bands is specifically associated to β-aggregated structures. *D* and *E*, transmission images representing IAV-infected lung slices observed at day 1 and 2. The *dots* represent the IR spectra acquired in the WT-infected lung slices presenting a β-aggregated structure signature. The color is associated to each IR spectrum depending on the score obtained by PCA (from *yellow* to *red*). *White dots* correspond to outlier spectra presenting a β-aggregated structure signature but discarded from the PCA because of the abnormal deformation of the baseline of the spectrum in order to avoid any misinterpretation. The scale bars represent 100 μm. FT-IR, Fourier-transform IR; IAV, influenza A virus; p.i., postinfection; PCA, principal component analysis.

Previously, we used multivariable statistical data analysis applied to sFT-IR spectroscopy to detect PB1-F2 β-aggregated structures at the single-cell level in IAV-infected cells (22). A specific signature corresponding to PB1-F2–aggregated amyloid-like fibers was defined in a cell-type–dependent and time-dependent manner. In this study, we applied the aforementioned method to investigate the presence of PB1-F2 β-aggregated structures in the lungs of IAV-infected mice expressing PB1-F2 within the bronchioles displaying the highest level of Trp fluorescence by sDUV microscopy. Using the serial cryosections previously mapped and analyzed by sDUV microscopy, we randomly acquired sFT-IR spectra within the bronchioles following straight lines from the outside (*i.e.*, the lumen) to the inside of the tissue (cells composing the bronchiole and intra-alveolar space). FT-IR spectra were processed as previously described, analyzed by principal component (PC) analysis, and represented as score plots and loading plots (22). Score plots (Fig. 2, *A* and *C* and Fig. S1*A*) enable us to plot the data in a form where each FT-IR spectrum is represented as a single point in a relevant space (PC). Then, loading plots (Fig. 2*B* and Fig. S1*B*) permit to enlighten the correlation between the variables (wave number values) and determine to what extent a variable is influencing the model of study (herein, IR signature of IAV-infected bronchioles). Finally, we localized each spectrum of interest on the bright field image of the studied bronchiole with a color code corresponding to the score calculated for each spectrum by the multivariate analysis (Fig. 2, *D* and *E* and Fig. S1*C*; maps of ΔF2-infected mice are presented in Fig. S2). FT-IR is a classical method to study the folding/unfolding and aggregation of proteins in particular, thanks to the amide I region (1715–1575 cm^−1^), which is very sensitive to protein secondary structure (22, 25, 26). Thus, we paid special attention to this region of the spectrum, which allows us to discriminate between different types of β-sheet structure signatures (27). Figure 2 displays the comparison between the secondary structure content of WT and ΔF2-infected mice at day 1 and day 2 p.i. (day 3 is presented in Fig. S1). At day 1, the score plot presented in Figure 2*A* revealed two independent clusters following the PC2 axis, which represents 31.7% of the total variance. The loading plot associated to PC2 allows us to determine that the main variation in the positive direction of PC2 (associated to WT-infected mice) is mainly because of peak variations with maxima situated at 1632 and 1668 cm^−1^. The peak at 1632 cm^−1^ is indicative of amyloid fibrils and aggregates. The second peak at 1668 cm^−1^ is ascribed to internal random coiled segments (disordered structures) and formation of β-turns. In contrast, the peak corresponding to the main variation in the negative direction of PC2 (associated to ΔF2-infected mice) at 1655 cm^−1^ is related to α-helical structures (28). Analysis of the spectra recorded at day 2 leads to the same observation: clear discrimination along the PC2 (31.7% of the total variance) axis between WT- and ΔF2-infected mice although the clusters are partially overlapping compared with day 1 (Fig. 2*C*). The two same main peaks were observed at 1633 and 1668 cm^−1^ in the positive values, and one peak at 1655 cm^−1^ was observed in the negative values of PC2 (Fig. 2*B*). Interestingly, a third peak appeared at 1691 cm^−1^ associated with WT-infected mice. The peak at 1691 cm^−1^ associated with the second peak at 1633 cm^−1^ is specifically ascribed to aggregated structures such as amyloid aggregates and strengthening the presence of β-aggregated structures in the bronchioles of PB1-F2 expressing–infected mice. This IR signature is similar to the PB1-F2 β−aggregated structures that have been determined at the single-cell level (22). A similar pattern was observed at day 3 (Fig. S1), although the score plot displays partially overlapping clusters following the PC1 axis (28% of the total variance). The loading plot presented in Fig. S1*B* indicates a pattern similar to what is observed at day 2, with two peaks at 1630 and 1690 cm^−1^ in the positive values and one peak at 1659 cm^−1^ in the negative values. The shift of the minor peak from 1633 to 1630 cm^−1^ in association with the major peak at 1690 cm^−1^ is indicative of highly aggregated β-sheet structures in WT-infected mice. In comparison with day 1 and 2, a new peak at 1643 cm^−1^ was observed at day 3, whereas the peak at 1668 cm^−1^ disappeared. It is very interesting to note that this domain of the spectrum (1630–1643 cm^−1^) is generally ascribed to native β-sheet structures, suggesting a phenomenon of accumulation of such structures in a time-dependent manner.

Thus, the comparison of spectra between WT- and ΔF2-infected mice at day 1, 2, and 3 enabled us to confirm the formation of β-aggregated structures at the major site of infection (*i.e.*, bronchioles) in the lungs of PB1-F2–expressing mice and a propensity of PB1-F2 amyloid-like structures to accumulate over time. The comparison over time allows us to observe the evolution of the IR signature during the course of the infection from native β-sheet structures to highly aggregated structures.

### PB1-F2 expression contributes to NF-κB induction

**Figure 3:**
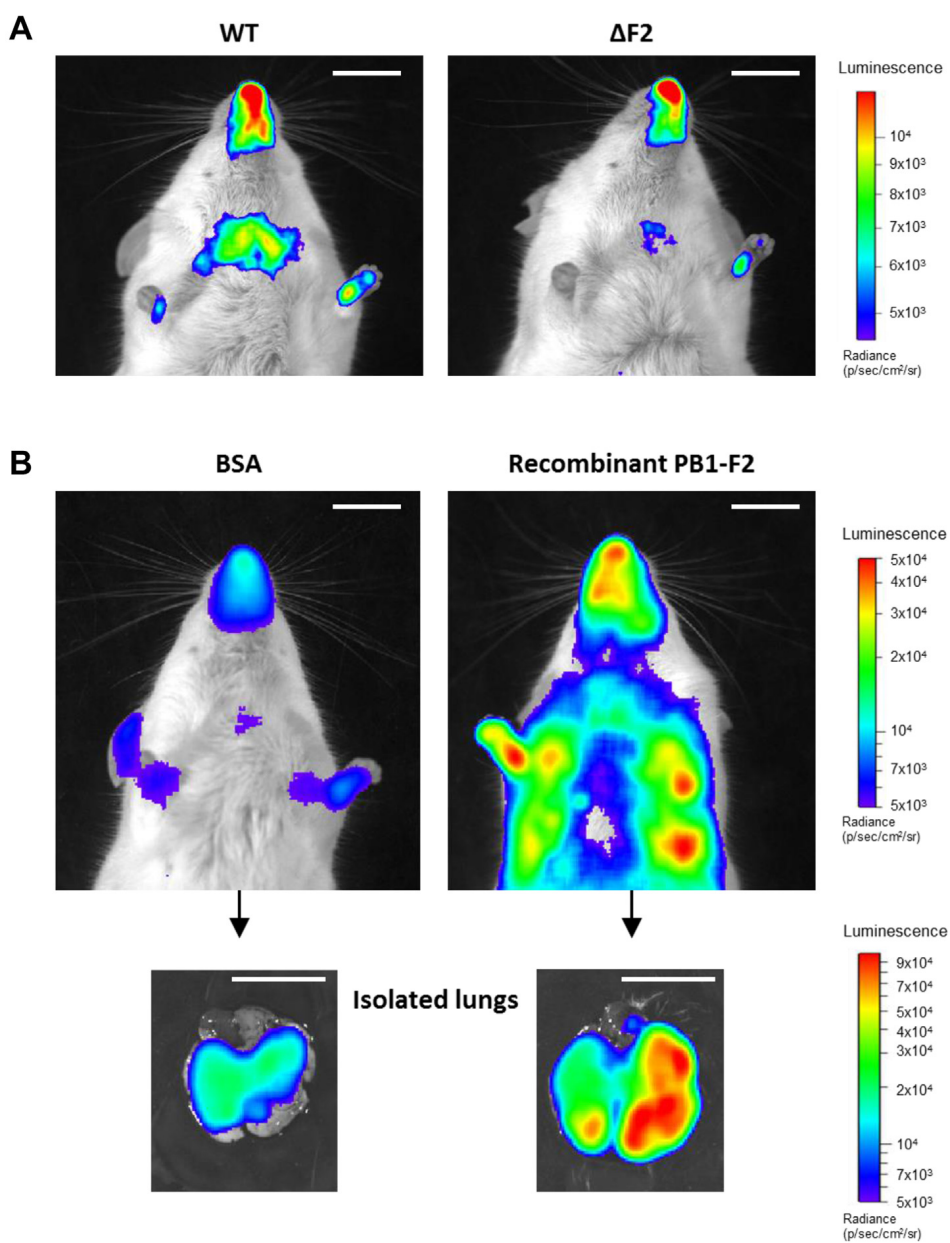
**PB1-F2 induces NF-κB activity in mice.***A*, groups of six NF-κB luciferase transgenic mice were infected with 1 × 10^4^ PFU of WT or ΔF2 IAV. Three days p.i., mice were anesthetized and luciferin was intranasally instilled (0.75 mg kg^−1^). Bioluminescence was then measured using the IVIS system. The scale on the *right* indicates the average radiance: the sum of the photons per second from each pixel inside the ROI/number of pixels (photons/s/cm^2^/sr). *B*, groups of six NF-κB luciferase transgenic mice were instilled with 50 nmol of BSA or 50 nmol of WSN rPB1-F2 for 18 h, and photon emission was then analyzed using the IVIS system. The mice were then euthanized and lungs extracted to estimate the tissue luminescence (*bottom panel*). The scale bar represents 1 cm. BSA, bovine serum albumin; IAV, influenza A virus; p.i., postinfection; ROI, region of interest.

Using NF-κB luciferase transgenic mice (8), PB1-F2 has been shown to increase the inflammatory state initially induced by the IAV infection (6). As confirmed in Figure 3*A*, a stronger NF-κB activity was induced in the lungs of WT IAV-infected mice at 3 days p.i. when compared with ΔF2-infected mice. In order to study the impact of PB1-F2 in the absence of infection, PB1-F2 was recombinantly expressed (rPB1-F2) to be delivered by intranasal instillation of NF-κB luciferase transgenic mice. Mice were instilled with a dose of 50 nmol of WSN rPB1-F2 (monomeric form) or of bovine serum albumin (BSA) as a control. The NF-κB luciferase signal of rPB1-F2–treated mice displayed an overwhelming intensity, suggesting a dysregulated response with subsequent hyperinflammation (Fig. 3*B*) compared with control conditions. To confirm the lung localization of the inflammatory process, the mice were euthanized and their lungs were extracted to monitor the luminescence emission. Figure 3*B* (*bottom panels*) clearly shows the PB1-F2–induced NF-κB activity in the lungs. This massive inflammation occurred with a very fast kinetic: 18 h postinstillation and was associated with signs of morbidity (hunched posture, ruffled fur, hypothermia, and lethargy). Ultimately, rPB1-F2–instilled mice died after 24 to 36 h postinstillation.

### NF-κB induction depends on the PB1-F2 sequence and oligomerization state

**Figure 4:**
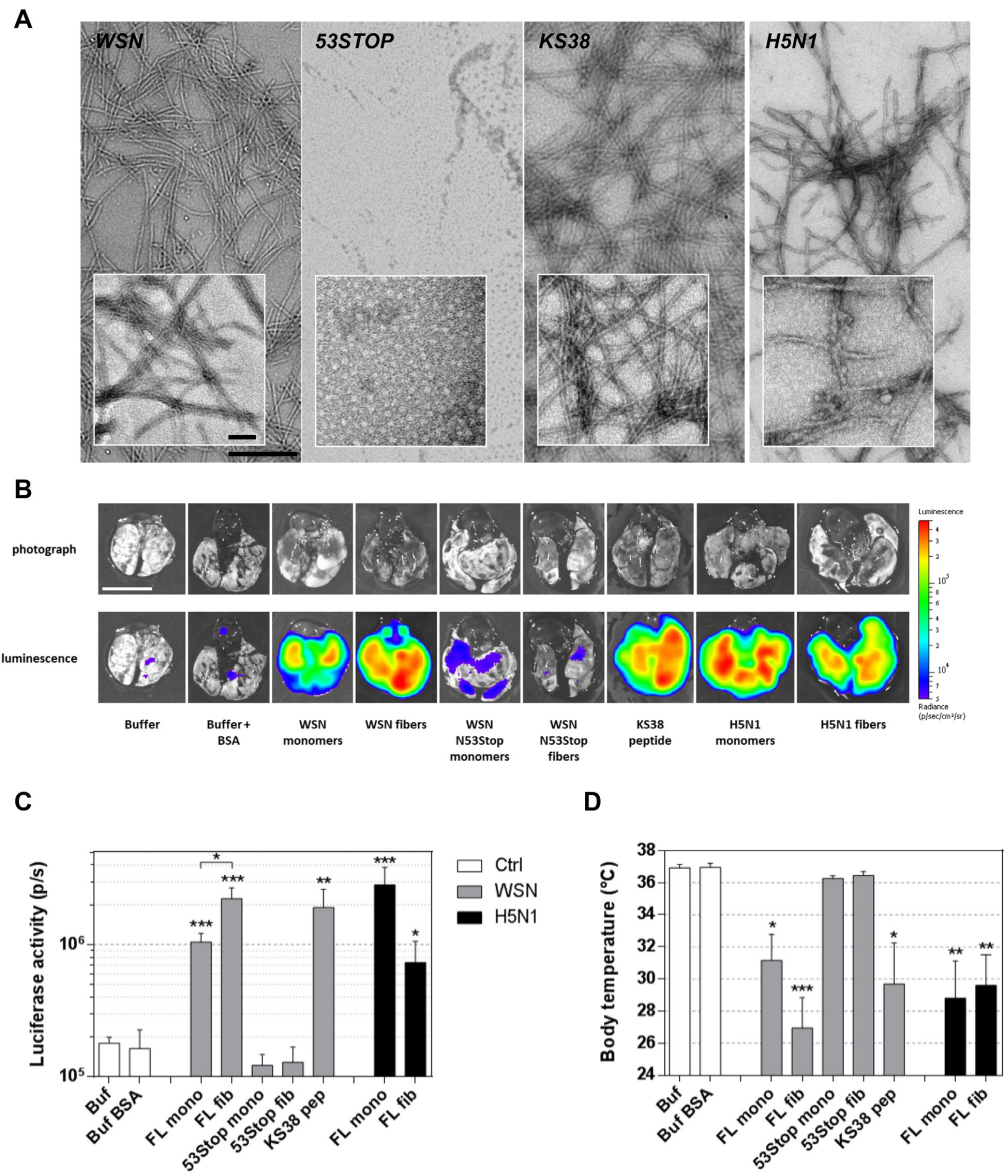
**PB1-F2 instillation induces lung inflammation.***A*, observation by electron microscopy of different preparations of recombinant PB1-F2 instilled into NF-κB transgenic mice: A/WSN/1933 (H1N1) fibers (WSN), aggregates formed by the peptide constituted by the 53 N-terminal residues of PB1-F2 WSN (53STOP), C-terminal PB1-F2 peptide fibers (KS38), and A/Duck/Niger/2090/2006 (H5N1) fibers (H5N1). The scale bars represent 50 and 500 nm. *B*, NF-κB luciferase transgenic mice were instilled intranasally with 50 nmol of different oligomerized forms of PB1-F2. At 18 h p.i., the mice were euthanized and the lungs extracted to monitor luminescence emission. The scale bar represents 1 cm. Bioluminescence was measured using the IVIS system. *C*, the luciferase signal was quantified using the Living Image 4.0 software. The scale on the *right* indicates the average radiance: the sum of the photons per second from each pixel inside the ROI/number of pixels (photons/s/cm^2^/sr). *D*, body temperature of instilled mice at 18 h p.i. Results are expressed as means ± SEM, and *p* values were calculated against buffer condition (∗*p* < 0.05; ∗∗*p* < 0.01; and ∗∗∗*p* < 0.001). p.i., postinfection; ROI, region of interest.

To further analyze and compare the impact of different oligomerization states of rPB1-F2, we instilled multiple forms of the protein: monomeric and prefibrillated forms of the full-length PB1-F2 (herein named FL WSN) or of its N-terminal part (53 amino acids; herein named 53Stop) and of the synthetic peptide corresponding to its C-terminal part (38 amino acids; herein named KS38) (Fig. S3). We have previously demonstrated that PB1-F2 monomers form amyloid-like oligomers capable of assembling into amyloid fibers. Moreover, PB1-F2–mediated cytotoxicity is mainly caused by amyloid-like oligomers (17). Therefore, two mechanisms of virulence could be expected: one mediated through cytotoxicity of oligomers, and the other induced by fibers recognition as pathogen danger signal. Given the importance of the sequence polymorphism of PB1-F2, we also used an rPB1-F2 protein originating from a highly pathogenic strain of avian H5N1 IAV (A/duck/Niger/2090/2006(H5N1)) (7, 9). While the full-length rPB1-F2 fibrillation was only induced in a hydrophobic environment as already shown (12), the KS38 peptide spontaneously displayed a high propensity to fibrillate (Fig. 4*A*). Indeed, we have previously shown that the KS38 peptide spontaneously folds into amyloid-like structures in neutral and basic aqueous solutions (17). As shown in the inset, WSN and H5N1 fibers tended to acquire a supramolecular organization specific of amyloid fibers, whereas KS38 fibers seemed to remain in a protofibrillar form. In contrast, the 53Stop peptide only formed small amorphous aggregates (18) (Fig. 4*A*). The different forms of rPB1-F2 were then instilled into NF-κB luciferase transgenic mice in order to quantify the amount of inflammation induced by full-length and truncated forms of PB1-F2, oligomerized or not. Figure 4*B* shows representative isolated lungs of each condition and demonstrates the proinflammatory effect of the different supramolecular forms of PB1-F2 with the exception of the 53Stop peptide that displays luminescence signal equivalent to background levels. Interestingly, the 53Stop form is the only PB1-F2 configuration that is not able to fibrillate. When using prefibrillated WSN rPB1-F2, we observed an enhanced inflammation compared with the monomer-treated mice (Fig. 4*C*). By contrast, when studying H5N1 PB1-F2, the inflammatory signal appears more important when using monomeric form of PB1-F2. Such a result suggests a very potent cytotoxic activity of the H5N1 monomers that overcomes the danger signaling exerted by prefibrillated H5N1 PB1-F2. Altogether, the results confirm that the level of inflammation in the lungs of NF-κB luciferase transgenic mice directly depends on the intrinsic capacity of the protein to form cytotoxic oligomers and aggregated amyloid-like fibers. More precisely, inflammation signal is observed after instillation of PB1-F2 able to oligomerize to finally form fibers, whereas the 53Stop peptide, unable to fibrillate, induced the lowest signal. Interestingly, this lung inflammatory response is inversely proportional to the body temperature of the instilled mice (Fig. 4*D*) and shows a very good correlation between inflammation and loss of temperature (Spearman *p* < 0.0001; Fig. S4). Finally, our data demonstrate that the domain of PB1-F2 involved in the exacerbation of the inflammatory response resides in the C-terminal part of the protein.

### PB1-F2 instillation provokes massive desquamation of the ciliated epithelium

**Figure 5:**
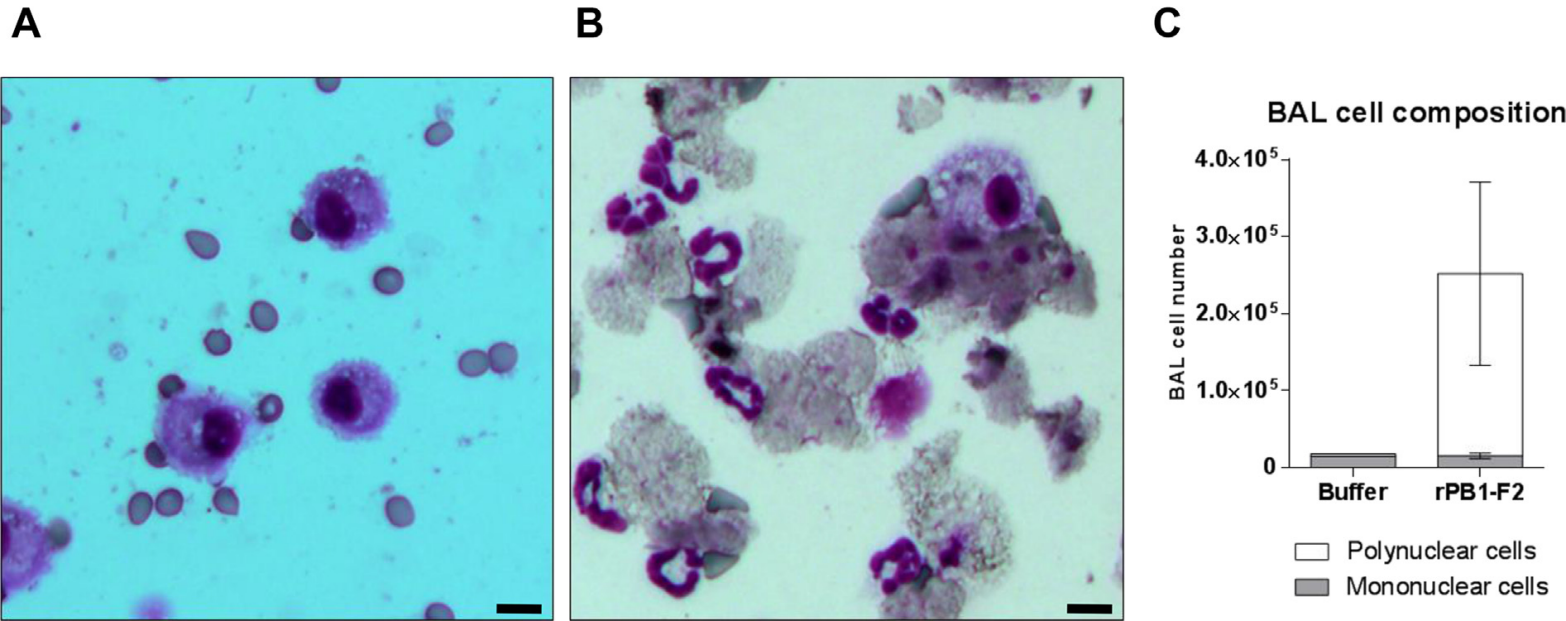
**PB1-F2 instillation induces epithelial desquamation and neutrophil recruitment.** BALB/c mice were intranasally instilled with 50 nmol of WSN rPB1-F2. At 18 h postinstillation, mice were euthanized and BAL fluids were collected. Cellular fractions of the BAL fluids were then purified, and 10^5^ cells were cytospinned to perform histological observation. *A*, cytospin obtained from a control mouse instilled with BSA. *B*, cytospin of a mouse instilled with PB1-F2. *C*, BAL fluid quantification of mononuclear and polynuclear cells. Results are expressed as means ± SD (the scale bar represents 20 μm). BAL, bronchoalveolar lavage; BSA, bovine serum albumin.

Given the potent proinflammatory activity of PB1-F2 observed in mouse lungs, we further studied the cellularity of bronchoalveolar lavage (BAL) fluids of PB1-F2–instilled mice. BAL fluid sampling is thought to reflect the degree of inflammation within and around the bronchial lumen and in deeper alveolar compartments (29). Under basal conditions, the cell constituents of BAL fluid are mainly composed of alveolar macrophages as shown in Figure 5*A*. As expected, when mice were instilled with rPB1-F2 (monomeric form), the cell composition of BAL fluids severely increased with a high number of neutrophils being recruited to the airways (Fig. 5, *B* and *C*). A close observation of the cytospin samples showed accumulation of debris and damaged nucleated cells. Remarkably, neutrophils were tightly associated to desquamated necrotic epithelial layers (Fig. 5*B*). It is well known that the bronchial–epithelial cell layer is extremely vulnerable to trauma; however, the effect of PB1-F2 on epithelial integrity appears to be very destructive and offers further evidence of the extremely deleterious impact of PB1-F2 on cellular structures.

### PB1-F2 instillation activates both inflammasome-dependent and inflammasome-independent innate immune responses

**Figure 6:**
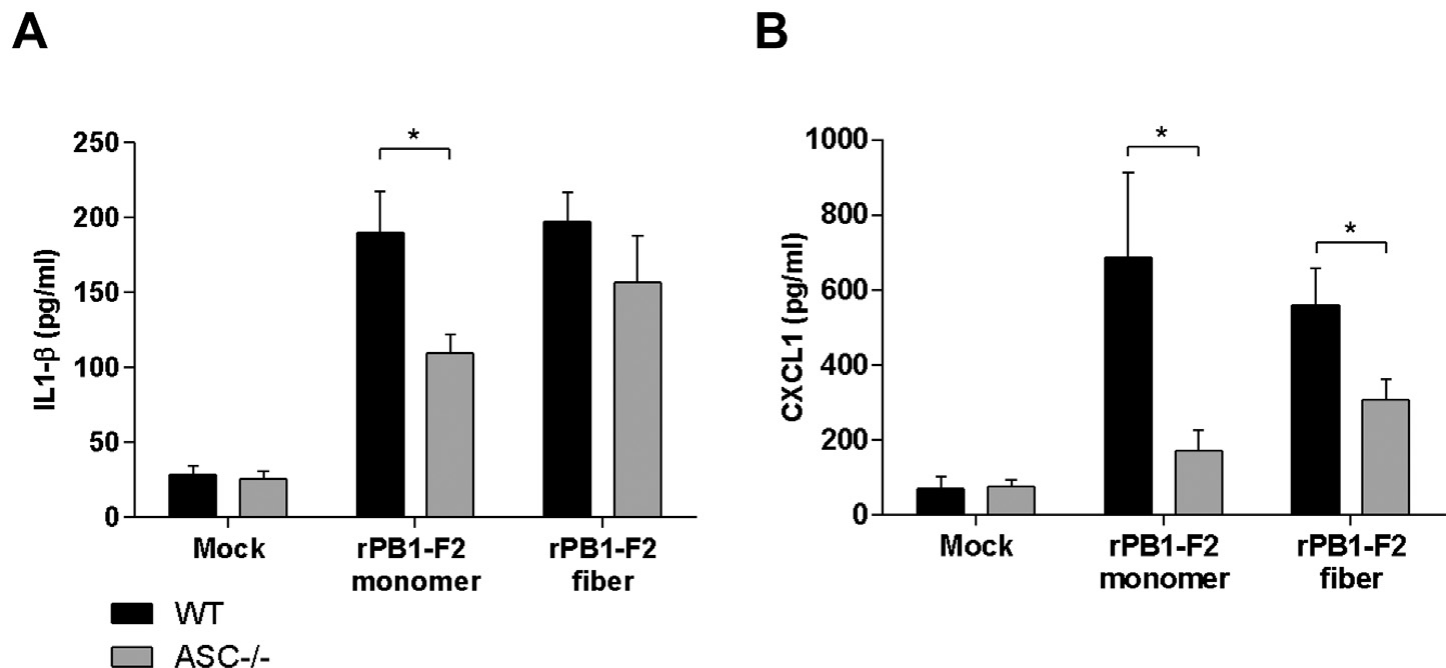
**PB1-F2 instillation induces cytokine production partly dependent on the inflammasome activity.** WT or ASC^−/−^ C57BL/6 mice were intranasally instilled with 50 nmol of monomeric WSN rPB1-F2 or the fibrillated form of WSN rPB1-F2. At 18 h p.i., mice were euthanized and BAL fluids were collected for cytokine quantification: (*A*) IL-1β and (*B*) CXCL1. Results are expressed as means ± SEM (∗*p* < 0.05). CXCL1, C-X-C motif chemokine ligand 1; IL-1β, interleukin-1β; p.i, postinfection.

It has been previously established that aggregated forms of PB1-F2 were recognized by NLRP3 (19). NLRP3 is a NOD-like receptor described for its aptitude to mount an innate immune response by triggering an inflammasome activation (30). NLRP3 signaling pathway is dependent on ASC (also known as PYCARD), a key adaptor protein of the inflammasome (31). Importantly, ASC^−/−^ mice are not able to mount a response upon NLRP3 activation. Interleukin-1β (IL-1β) synthesis, maturation, and secretion is dependent on inflammasome activation, whereas ASC is not required for the secretion of C-X-C motif chemokine ligand 1 (CXCL1) (KC). Thus, we have chosen these two cytokines because they are highly induced during IAV infection and because they represent inflammasome-dependent (IL-1β) and inflammasome-independent cytokine (CXCL1). In view of this consideration and to better understand the role of the inflammasome signaling in our mouse model of PB1-F2–mediated pathogenesis, we assessed BAL fluid cytokine content in WT and ASC^−/−^ mice instilled with 50 nmol of WSN rPB1-F2 (Fig. 6). About 18 h postinstillation, mice were euthanized, and BAL fluids were collected to quantify the amount of IL-1β and CXCL1. Figure 6, *A* and *B* represents the data obtained when assaying IL-1β and CXCL1, respectively. The amounts of cytokines measured within BAL fluids of monomer-treated mice revealed a partial inflammasome dependence of IL-1β and CXCL1 secretions. However, when the fiber form of rPB1-F2 was used to instill the mice, this inflammasome dependency was not observed for IL-1β and only partially seen for CXCL1. Based on these intriguing results, it appears that the pathological mechanism mediated by PB1-F2 is more complex than an inflammatory response exacerbation. The inflammasome machinery is clearly implicated in the sensing of PB1-F2; nevertheless, mice bearing an inflammasome deficiency still secrete a massive amount of inflammatory mediators.

### PB1-F2 alters respiratory functions independently of the inflammasome activation

**Figure 7:**
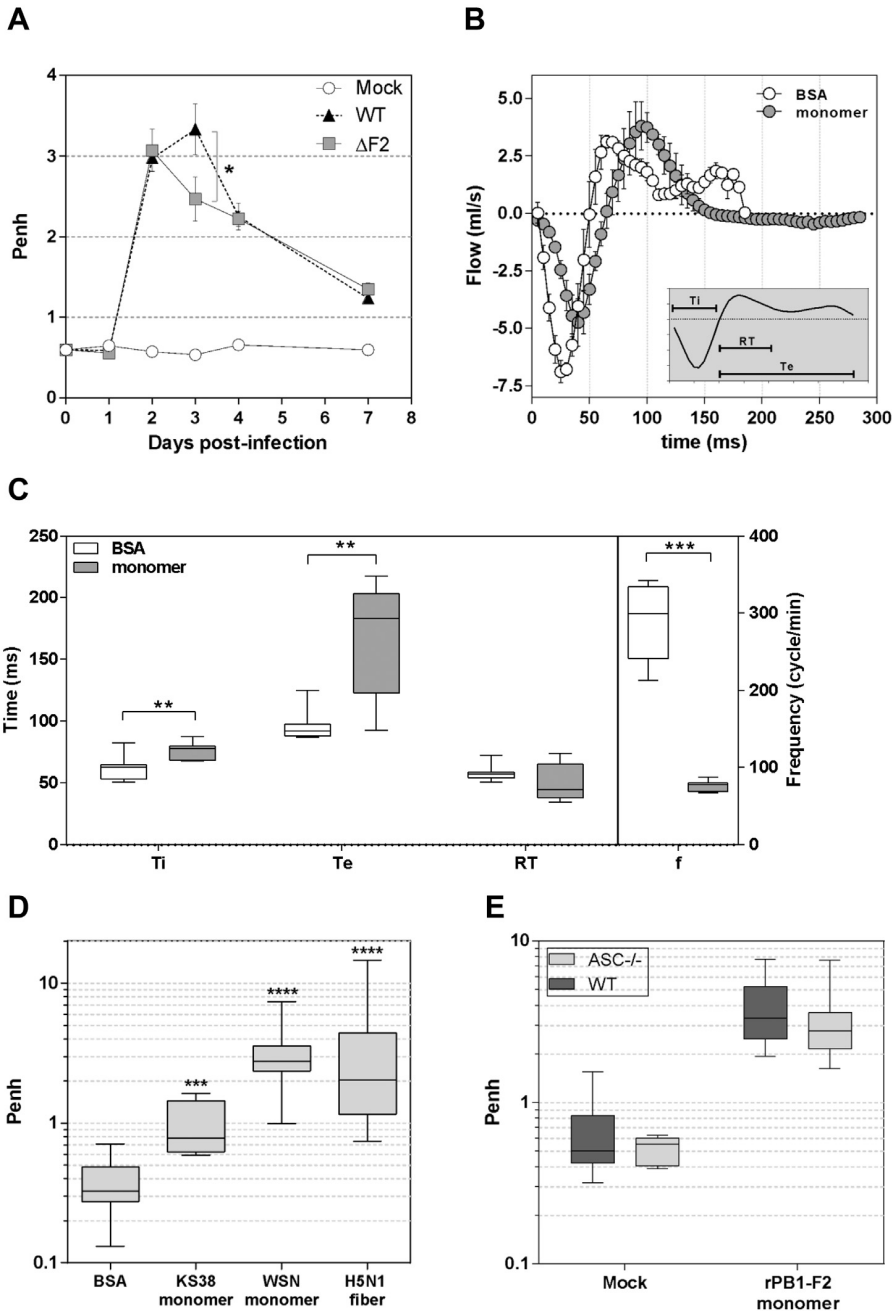
**PB1-F2 negatively impacts the respiratory parameters of infected and instilled mice.***A*, groups of six mice were mock infected or either infected by WT or ΔF2 WSN. Each mouse was then monitored by whole-body plethysmography in order to compute the unitless Penh index at days 0, 1, 2, 3, 4, and 7 p.i. *B*, representative respiratory cycles of instilled mice. *Open circles*: instillation with 50 nM BSA, *dashed circles*: instillation with 50 nM monomeric WSN rPB1-F2. *C*, representation of time of inspiration (Ti), time of expiration, relaxation time (RT), and frequency of breath of instilled mice. The 25th to 75th percentiles are represented by the box plots, with the horizontal bar indicating the mean value. Error bars indicate the standard deviation of measurements (∗∗*p* < 0.01; ∗∗∗*p* < 0.001). *D*, Penh index of instilled mice. Mice were instilled either with 50 nM BSA, 50 nM of KS38 peptide, 50 nM of monomeric WSN rPB1-F2, or 50 nM of fibrillated H5N1 rPB1-F2. After 18 h postinstillation, mice were monitored for respiratory parameters using whole-body plethysmography, and Penh index was calculated. *E*, comparison of Penh index of WT and ASC^−/−^ C57Bl/6 mice 18 h postinstillation of 50 nM BSA (mock) or 50 nM monomeric WSN rPB1-F2. BSA, bovine serum albumin; p.i., postinfection.

To go further into the characterization of the host response triggered by PB1-F2, we functionally explored the respiratory capacities of WT and ASC-deficient C57Bl/6 mice during IAV infection or rPB1-F2 instillation. We used whole-body plethysmography to measure the respiratory metric called enhanced pause (Penh), a unitless index of airway resistance (32). After infection, the Penh rapidly increased 2 days p.i. to reach a peak at day 3 p.i. in IAV-infected WT mice (Fig. 7*A*). This peak of Penh corresponds with the maximum expression level of PB1-F2 (6, 15, 33). Interestingly, when using the isogenic virus unable to produce PB1-F2, the Penh kinetic was significantly different, with a peak at day 2 p.i. followed by a rapid decrease at day 3, showing a statistically significant difference with the WT virus (Fig. 7*A*). These results suggest that PB1-F2 expression during IAV infection exacerbates the respiratory distress initially induced by viral replication. To further explore this hypothesis, we followed respiratory functions of mice instilled with rPB1-F2. As shown in Figure 7*B*, the respiratory cycles were modified by PB1-F2 including a reduced amplitude of inspired volume and an increased time of expiration. We compared the different times of inspiration, expiration, and relaxation of respiratory cycles and clearly observed a difference between BSA- and rPB1-F2–instilled mice (Fig. 7*C*). These differences impacted the breath frequency and, as a consequence, the volume of respired air (Fig. S5). We then compared the effects of the different forms of PB1-F2 on the Penh. Surprisingly, while WSN monomers and H5N1 fibers induced a comparable respiratory distress, the KS38 peptide induced moderate respiratory distress. This observation indicates that inflammatory activity and respiratory distress are not necessarily linked in the case of PB1-F2 (Fig. 7*D*). Finally, we explored the role of the inflammasome in the PB1-F2–induced airway resistance. However, as shown in the Figure 7*E*, the ASC^−/−^ mice exhibited similar Penh values than the WT mice, excluding any inflammasome implication in the respiratory distress mediated by PB1-F2.

## Discussion

In this work, we report for the first time the correlation between the supramolecular structure of PB1-F2, its expression mapping, and the resulting pathological disorders in an animal model of IAV infection. PB1-F2 appears as an IAV virulence factor responsible for disrupting the functionality of airways. Multiple lines of evidence suggest that PB1-F2 may mediate pathogenicity through two independent mechanisms. These two mechanisms are the direct consequence of the oligomerization and the fibrillation of PB1-F2. On one hand, PB1-F2 fibers are recognized by the inflammasome machinery (NLRP3) as a danger signal; and on the other hand, the aggregated fibers exhibit an important toxicity within the airways. The first mechanism, which implicates the inflammasome, will exacerbate the inflammatory response initially induced by IAV replication, suggesting that PB1-F2 participates to the deleterious and uncontrolled inflammatory pattern observed during acute IAV infection (34, 35). The second mechanism is independent of the inflammasome and clearly related to the oligomerization of PB1-F2, a mechanical process that appears to be highly detrimental for the targeted tissue. This process alters the epithelium integrity of the airways and, consequently, negatively impacts the respiratory capacities of the infected host, compromising its survival.

Taking advantage of our previous experience with synchrotron beamlines, we were able to detect the presence of PB1-F2–aggregated fibers within lungs of IAV-infected mice and to ascribe a specific IR signature to it. In our previous synchrotron radiation experiment combining the FT-IR and DUV microspectroscopies, we have determined a β-aggregated IR signature associated with the presence of PB1-F2 amyloid-like fibers in two IAV-susceptible cell types (U937 and A549 cell lines) in a time-dependent manner at the single-cell level (22). Importantly, the amyloid signature was only detected in immune cells (U937 cells), and not in epithelial cells (A549 cells), which displayed only a native β-sheet IR signature. Moreover, we have previously achieved the monitoring of the oligomerization of PB1-F2 in these two cell lines using a biosensor based on immobilized antibodies specific for the soluble monomeric or oligomeric forms of PB1-F2 (15, 16). Our results have demonstrated that the monomeric PB1-F2 accumulates from the early stage of the infectious cycle in both cell types and forms β-soluble oligomers in a time- and cell-dependent fashion. The final step implicating accumulation of PB1-F2 aggregates was only observed in IAV-infected U937 cells. Herein, we also demonstrate that such structures accumulate over time in IAV-infected lungs of mice, corroborating the results obtained at the single-cell level to those obtained at the tissue level (6, 11, 15, 16, 22).

Synchrotron radiation experiments allowed us to localize PB1-F2–specific β-aggregated structures within the bronchioles of infected mice. Such β-aggregated structures are not visible when studying the PB1-F2 knockout virus. At the beginning of an IAV infection, bronchi and bronchioles represent the major sites of infection. Hence, these β-aggregated structures accumulate within the tissue providing air passage to the alveoli. Given the proinflammatory effect of PB1-F2, such an accumulation will exacerbate leukocyte recruitment and alter mucus production (6, 8). In addition, smooth muscles lining the bronchioles will also be altered, and the contractibility of bronchioles will be reduced, inducing a loss of control in the air flow. PB1-F2 β-aggregated structures are therefore clearly implicated in the pathological process mediated by IAV infection.

Thereafter, we studied the impact of rPB1-F2 alone and tried to correlate the different forms of instilled PB1-F2 (in length and supramolecular organization) with the physiopathology of the infection. To this end, we instilled a dose of 50 nmol of recombinant PB1-F2 per mouse. This concentration is about 12 times higher than what is observed during an IAV infection and therefore could be considered as biologically irrelevant. We previously used surface plasmon resonance immunosensor to quantify the amount of PB1-F2 within infected mice. The concentration of PB1-F2 was estimated to be around 26 pmol per mg of lung tissue at day 4 p.i. (6, 33). Extrapolated to an entire mouse lung (*i.e.*, 150 mg), we can estimate the amount of PB1-F2 per lung to be around 4 nmol per lung during the early steps of the infection. However, surface plasmon resonance allows us to estimate only the soluble fraction of PB1-F2 and leads to an underestimation of the total amount of PB1-F2 per lung considering that the protein accumulates as aggregates overtime. Moreover, we believe that during infection, PB1-F2 is probably concentrated into microdomains (mainly within membranes) and, consequently, its concentration must reach amounts close or superior to concentrations used in our study. It is highly likely that once PB1-F2 reaches a certain concentration within cell membranes, it induces a desquamation of the infected epithelial cells as observed in Figure 5. Using 50 nmol, we were able to achieve this critical concentration, whereas instillation of the recombinant protein spreads the protein throughout the tissue instead of concentrating it in microdomains. The inflammatory effects on the mice are consequently more important than during viral infection. The desquamated cells observed in our report exhibited a degenerating appearance, with large clumps, smaller cell size, and abnormal nuclear staining compared with those in uninfected controls (Fig. S6). The general aspect of the desquamated cells bears some resemblance with necrotic cells, whereas PB1-F2 is usually described as an apoptosis inducer (36). However, the proapoptotic capacities of PB1-F2 have mainly been described in immune cells and not in epithelial cells. Interestingly, using acridine orange staining, a marker of necrosis, we have previously shown that PB1-F2 oligomers increase cell death at later stage of infection in IAV-infected cells. Once again, immune cells displayed a stronger staining than epithelial cells, suggesting that cell necrosis could be triggered by the accumulation of PB1-F2–aggregated amyloid-like fibers (17). Epithelial desquamation decreases the mucociliary clearance process efficiency and is thus consistent with the previously observed bacterial superinfection associated with PB1-F2 expression (5). In addition, PB1-F2–induced desquamation of the tracheobronchial epithelium may facilitate virus spreading to adjacent airway epithelium and also gives access to deeper regions of the lung. By reaching the lower respiratory tract, the virus prolongs its shedding time within the infected host, and consequently, maximizes its probability to infect new hosts by horizontal transmission. This could explain why PB1-F2, despite potent proinflammatory properties, is largely conserved in many human IAV strains.

The use of rPB1-F2 and of its associated peptides, PB1-F2 N-(53STOP) and C-(KS38) terminal peptides, and our expertise to produce the different oligomerized forms of PB1-F2 provided us the opportunity to decipher the implication of the different domains of the protein in the physiopathology of the infection. Indeed, the lack of PB1-F2 expression or the expression of truncated forms missing the C-terminal domain (considered as nonfunctional) was associated with an attenuation of the virus. Herein, we demonstrated that KS38 is highly associated with the propensity of the protein to oligomerize and form amyloid-like fibers, and we correlated this observation with an exacerbation of the inflammation and a disruption of airway function. As presented in Fig. S3, the key sequences and polymorphisms suggested or demonstrated to be involved in inflammation, cell death, cytotoxicity, exacerbation of the virulence, and susceptibility to secondary bacterial infections are located in the KS38 peptide (5, 6, 37). Interestingly, WSN and H5N1 PB1-F2 peptides used in this study differ only at one position of the four residues constituting the proinflammatory/anti-inflammatory motif (L/P_62_R/H_75_R/Q_79_L/S_82_). The H5N1 KS38 peptide, which contains the integral proinflammatory motif (**L**RRL), induced a stronger inflammation than the WSN KS38 peptide, which exhibits only a partially proinflammatory motif (**P**RRL). Structurally, PB1-F2 belongs to the intrinsically disordered family of proteins capable of undergoing a conformational transition from their native disordered state to α-helical or β-sheet conformations. The prediction of the presence of an α-amphipathic helix and of the peptide responsible for the oligomerization (13) and the formation of amyloid-like fibers (38) was also proposed within the sequence of KS38 peptide. The transition mechanism, and as a consequence, the oligomerization mechanism was shown to be triggered by different factors such as environmental conditions (pH (17), membrane composition (17, 18), and the environmental conditions (22)). Hence, the full-length monomeric PB1-F2 is soluble at pH 5 in solution but aggregates to amorphous structures displaying no cytotoxicity at physiological pH. In contrast, 53STOP peptide oligomerizes at acidic pH and exhibits no toxicity, whereas KS38 spontaneously oligomerizes and fibrillates at physiological pH and is highly cytotoxic (17, 39). Interestingly, the mucus is naturally slightly acidic (between 5.5 and 6.5 depending on the region of the respiratory tract), and the acidification of the airways can be considered as a marker of disease notably in the case of bacterial infection of the endobronchial region (40). Thus, it is tempting to hypothesize that pH- and membrane-dependent PB1-F2 oligomerization and cytotoxicity, which is herein illustrated by airway epithelium desquamation, could be correlated with the facilitation of secondary bacterial infections.

By disrupting the tracheobronchial epithelium, PB1-F2 alters the respiratory parameters of the infected host and induces respiratory distress, a pathological process classically quantified by measuring the Penh. Penh assesses the bronchial responsiveness and depicts an index of bronchoconstriction. Therefore, considering the high degree of bronchial tropism by IAV, Penh represents a very convenient and relevant readout when studying influenza-associated pathology (41, 42). In our assays, PB1-F2 instillation increases the mean inspiration and expiration times. The breath frequency is thus decreased and, as a result, the minute volume of inhaled air is reduced from 55 to 37 ml/min (Fig. S5). Such compromised breathing functions have dramatic consequences on the whole-body physiology of the infected host. The first consequences being the establishment of hypoxia within the altered lung, and as a result, a decrease in the arterial blood oxygenation (43) that induces potential hazardous effects on extrapulmonary organ structure and function. In this regard, it would be of great interest to characterize whether a hypoxia gene signature is associated with the lungs of rPB1-F2–instilled mice.

## Experimental procedures

### Viruses

Influenza A/WSN/1933 (H1N1) virus and its mutant unable to express the PB1-F2 protein were produced using the eight-plasmid reverse genetic system as previously described (6, 11, 12, 44).

### Mice infection

Female Balb/c mice (n = 105) and C57BL/6 (n = 31) were purchased from the “Centre d’Elevage R. Janvier” and were used at 8 weeks of age. ASC^−/−^ C57BL/6 mice (n = 32) were obtained from “CNRS UPS44 - TAAM - Transgénèse et Archivage d'Animaux Modèles”. NF-κB luciferase transgenic Balb/C mice (n = 74) were obtained by backcrossing NF-κB luciferase Transgenic B10.A (kind gift of Prof. Richard Flavell, Howard Hughes Medical Institute) with Balb/C mice to ensure the production of transgenic mice with white fur to avoid absorption of light by the dark skin and fur of the B10.A mice. Mice were housed in negative pressure isolators in a containment level 2 facility. Food and water were available *ad libitum*. Viral infection and sample collection were performed as previously described (34, 45). Mice were lightly anesthetized with a mixture of ketamine and xylazine (60 and 12 mg/kg, respectively) to minimize suffering and were inoculated intranasally with 1 × 10^6^ or 1 × 10^4^ PFU of virus in 50 μl PBS. Weights and rectal temperatures (BIO-TK9882; Bioseb Instruments) were recorded daily. Humane endpoints were used during infectious study: mice were euthanized by cervical dislocation when body weights were reduced to 80% of the starting weights or in case of a nontransient decrease in whole-body temperature <32 °C. In addition, animals that reached moribund state (unresponsive and unaware of stimuli) were also euthanized. Control mice for the infection received the same treatment as infected mice with the exception of the addition of virus in the infection mixture.

Infections were stopped at day 1, 2, and 3, and extracted lungs of mock- or IAV-infected mice were incubated in 4% paraformaldehyde solution and then rinsed in 70% ethanol before being stored at −80 °C until sectioning. Frozen tissues were cut into 8 μm-thick sections with a cryotome (Leica) at −35 °C. The samples of tissue were not submitted to any other treatment (mounting medium, optimal cutting temperature, or gelatin) to avoid introducing any bias in the FT-IR analysis. Cryosections were fixed on zinc selenide 4-mm diameter IR transparent window or on 25-mm diameter quartz suprasil coverslips for sFT-IR or sDUV microscopy, respectively.

### PB1-F2 protein purification and instillation

PB1-F2 protein of A/WSN/1933 (H1N1) influenza virus was expressed and purified as described previously (12). Briefly, the gene encoding either full-length PB1-F2(1–90) protein or the N-terminal domain of PB1-F2(1–52) (Nter) were cloned into the pET22b+ expression vector (Novagen) to express His6-tagged protein versions. Transformed competent BL-21 Rosetta cells (Stratagene) were incubated with 1 mM isopropyl-β,D-thiogalactopyranoside for 4 h at 37 °C. After cell lysis and solubilization in 8 M urea buffer, the recombinant PB1-F2-His proteins were purified from inclusion bodies on a Hitrap-IMAC column using the AKTA Purifier-100 FPLC chromatography system (GE Healthcare). Fractions collected containing PB1-F2-His proteins were further purified by size exclusion chromatography on a 120 ml Superdex 200 column. Urea was removed on a G25 desalting column equilibrated with 5 mM ammonium acetate buffer (pH 5). PB1-F2-His proteins were lyophilized and stored at −20 °C. The C-terminal domain of PB1-F2(53–90) (pCter) was custom made by Proteogenix. Prior to instillation, lyophilized protein powder was dissolved in 50 mM sodium acetate at pH 5 buffer, and its concentration was determined by measuring absorbance at 280 nm using extinction coefficient deduced from its composition of 28,990, 5500, and 4833 M^−1^ cm^−1^ for PB1-F2(1–90), PB1-F2(1–52), and PB1-F2(53–90), respectively. PB1-F2 fibers were produced as previously described (17). PB1-F2 was incubated with 0.01% (w/v) SDS in 5 mM sodium acetate buffer (pH 5) during 45 min at room temperature. For instillation, mice were lightly anesthetized with a mixture of ketamine and xylazine (60 and 12 mg/kg, respectively) and were intranasally instilled with 50 nmol of PB1-F2 in 50 μl of 5 mM sodium acetate at pH 5. Control mice were instilled with 5 mM sodium acetate at pH 5 alone or complemented with 50 nmol of BSA.

### Synchrotron DUV fluorescence imaging

The full-field synchrotron DUV imaging system is constructed around a Zeiss Axio Observer Z1 (Carl Zeiss) inverted microscope constructed with quartz-only optics. The white beam of DISCO beamline at the SOLEIL Synchrotron (46) is monochromatized by an iHR320 (Horiba) before coupling with the entrance of the modified Zeiss Axio Observer Z1, the monochromatic beam was settled at 280 nm to determine the level of Trp and tyrosine in cells. A sharp dichroic mirror transmitting only above 300 nm (Omega Optical) reflected the incident light before focalization onto the sample through a Zeiss Ultrafluar ×40 (numerical aperture 0.6, glycerine immersion) objective (47, 48). Emission was recorded with a Pixis 1024-BUV (Princeton Instruments) camera after passing through a series of bandpass filters (Semrock). A mosaic mode acquisition (slide explorer from μManager) was used to scan the slice in a low-resolution mode, each picture composing the mosaic corresponding to 150 × 65 μm^2^ (more than 1000 pictures/map). Power measured on the sample was close to μW/cm^2^. The whole system was controlled *via* μManager (49). ImageJ software (50) was used to quantify the Trp/tyrosine fluorescent intensity of each ROI and to calculate the mean fluorescence normalized to the surface area of the ROI.

### Synchrotron FT-IR microspectroscopy

sFT-IR microscopy was performed at the SOLEIL synchrotron (Gif-sur-Yvette) using the SMIS beamline (SOLEIL Synchrotron). All spectra were recorded in transmission mode on a Nicolet Continuum XL microscope (Thermo Fisher Scientific). The microscope comprises a motorized sample stage and a liquid nitrogen-cooled mercury cadmium telluride detector (50 μm element size). The microscope operates in confocal mode using a ×32 infinity corrected Schwarzschild objective (numerical aperture = 0.65) and a matching ×32 condenser. All IR spectra were recorded using a dual mask aperture of 10 × 10 μm^2^. Individual spectra were saved in log (1/R) format at 4 cm^−1^ spectral resolution, with 128 coadded scans encompassing the mid-IR region from 4000 to 800 cm^−1^. All IR spectra were preprocessed and submitted to multivariate data analysis (Matlab, version 9.5; Mathworks). Spectra showing a strong baseline distortion because of dispersion artifact have been carefully removed and classified as outlier spectra. Second derivatives of the spectral data were assessed (nine-point Savitzky–Golay filter) to enhance the spectral resolution of the absorption bands and remove baseline variations inherent to the analytical environment and the optical or physicochemical properties of the heterogeneous samples. The second-derivative IR spectra were unit vector normalized and mean centered before analyzed by PC analysis. The computation of PCs was based on the nonlinear iterative projections by alternating least squares algorithm. Although the score plots allowed a comparison of IR spectra, the corresponding loading plots revealed the main characteristic absorption bands.

### *In vivo* luminescence measurements

Bioluminescence measurements of the NF-κB luciferase transgenic mice were measured using the IVIS 200 imaging system (PerkinElmer). Mice were anaesthetized, and luminescence was measured 5 min after intranasal injection of 50 μl of PBS containing d-luciferin (0.75 mg kg^−1^; Sigma). Living Image software (version 4.0; PerkinElmer) was used to measure luciferase activities. Bioluminescence images were acquired for 1 min with f/stop = 1 and binning = 8. A digital false-color photon emission image of the mouse was generated, and photons were counted within the whole-body area. Photon emission was measured as radiance in p.s^−1^.cm^−2^.sr^−1^.

### ELISA

CXCL1 (KC) and IL-1β concentrations in mice BAL fluids were determined using DuoSet ELISA kits obtained from R&D Systems.

### Plethysmography

Respiratory measurements were acquired by using a whole-body plethysmograph (EMKA). Briefly, vigil mice were placed in chambers that allow measurement of the differential pressure within the chambers because of breathing of the animal. Following acclimation, mice were measured for a 5-min period. Lung function parameters that were analyzed included enhanced pause (Penh). Penh is a derived measure of respiratory distress and has been shown to be an excellent parameter to assess and describe respiratory pathogenesis of IAV-infected mice (43, 51, 52).

### Statistical analysis

Luminescence measurements, body temperature, leukocyte counts, ELISA, and quantification of respiratory parameters are expressed as the mean ± SEM of at least three separate replicates, and statistical analyses were performed using the Student's *t* test for pairwise comparisons and ANOVA for multiple comparisons.

### Ethics statement

This study was carried out in accordance with INRAE guidelines in compliance with European animal welfare regulation. The protocols were approved by the Animal Care and Use Committee at the “Centre de Recherche de Jouy-en-Josas” (COMETHEA) under relevant institutional authorization (“Ministère de l’éducation nationale, de l’enseignement supérieur et de la recherche”), authorization number: 2015100910396112v1 (APAFIS#1487). All experimental procedures were performed in a biosafety level 2 facility.

## Data availability

All data are contained within the article.

## Supporting information

This article contains supporting information (23, 53, 54, 55, 56, 57, 58).

## Conflict of interest

The authors declare that they have no conflicts of interest with the contents of this article.
