# Supplementary material for: PB1-F2 amyloid-like fibers correlate with proinflammatory signaling and respiratory distress in influenza-infected mice
Source: J Biol Chem. 2021 Jun 17;297(1):100885. doi: 10.1016/j.jbc.2021.100885 (PMC8294585; doi:10.1016/j.jbc.2021.100885)
Supplement: Supplemental Figures S1–S6 [file mmc1.pdf]

Molecular characterization of PB1-F2-mediated pathology during *in vivo* influenza infection in mice.

Christophe Chevalier[1¶#], Olivier Leymarie[1#], Laura Sedano[1], Bruno Da Costa[1], Charles-Adrien Richard[1], Pauline Maisonnasse[1], Matthieu Réfregiers[2], Frédéric Jamme[2], Ronan Le Goffic[1¶]

[1]VIM, INRAE, Université Paris-Saclay, 78350, Jouy-en-Josas, [2]Synchrotron SOLEIL, L'Orme des Merisiers, 91190 Saint-Aubin, Gif-sur-Yvette, France.

<u>List of Supporting Material :</u>

1. Supplementary figure 1: Synchrotron FT-IR microspectroscopic cartography of secondary structures content of WT and ΔF2-infected IAV-infected lung slices at day 3 p.i. within the amide I region.
2. Supplementary figure 2: Transmission Image representing ΔF2-infected lung slice observed at day 1,2 and 3.
3. Supplementary figure 3: Alignment of PB1-F2 sequences from A/WSN/1933(H1N1) and A/duck/Niger/2090/2006(H5N1).
4. Supplementary figure 4: Correlation of the luciferase activity (*i.e.* NF-kB activity) with body temperature of PB1-F2-instilled mice 18 h post-instillation.
5. Supplementary figure 5: Minute ventilation of PB1-F2-instilled mice.
6. Supplementary Figure 6: Ciliated epithelial cells.

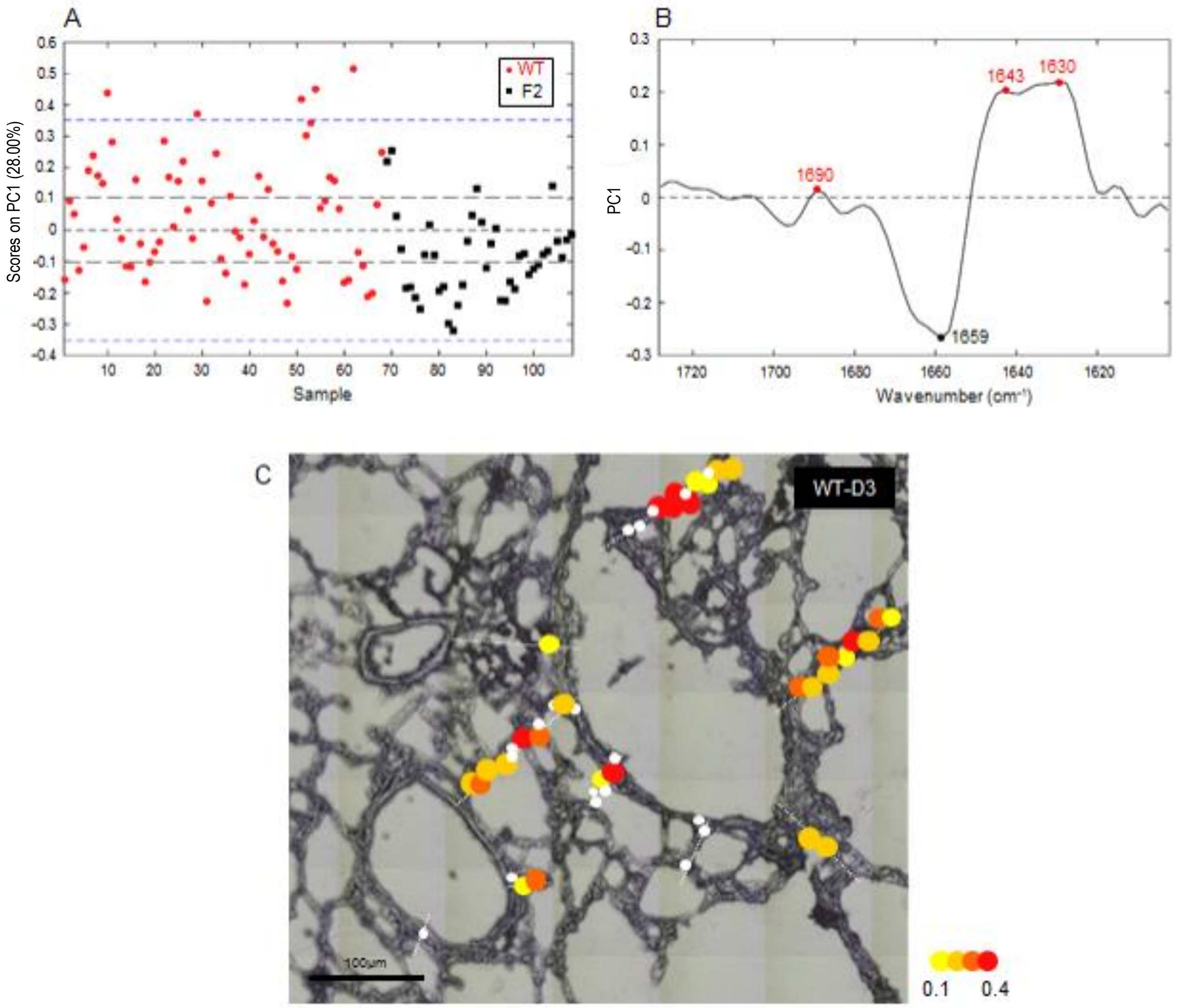

Supplementary figure 1: Synchrotron FT-IR microspectroscopic cartography of secondary structures content of WT and ΔF2-infected IAV-infected lung slices at day 3 p.i. within the amide I region.
(A) Score plot of principal component (PC) analysis from the 1700-1600 cm^{-1} band IR spectra. The explained variance for PC1 is 28% at day 3. Red circles and black squares correspond to single IR-spectra recorded in lung slices of mice infected with virus expressing PB1-F2 (WT) or with PB1-F2 knocked-out virus (ΔF2), respectively. **(B) Loading plot linking the variable space and PC sub-space (PC1).** At day 3, PCA score plot showed that the WT and F2 groups are separated along PC1 (A). The same signature specifically associated to β-aggregated structures was observed at day 3 with two peaks at 1630-1690 cm^{-1} (β-aggregated structures). A remarkable shift of the peak at 1668 cm^{-1} to 1643 cm^{-1}, attributed to a switch between unordered β-sheet structures to native β-sheet structures respectively, was also observed. **(C) Transmission image representing an IAV-infected lung slice observed at day 3 p.i.** The dots represent the IR spectra presenting a β-aggregated structure signature which were acquired in the WT-infected lung slice. The color is associated to each IR spectrum depending on the score obtained by principal component analysis (from yellow to red). White dots correspond to outlier spectra presenting a β-aggregated structure signature but discarded from the PCA because of the abnormal deformation of the baseline of the spectrum in order to avoid any misinterpretation. Scale bar = 100 µm.

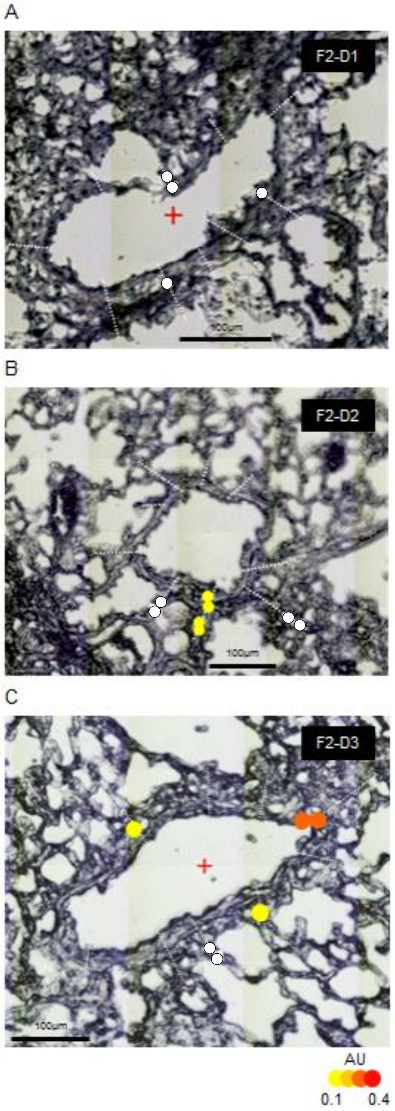

Supplementary figure 2: Transmission Image representing ΔF2-infected lung slice observed at day 1,2 and 3.

The dots represent the IR spectra acquired in the ΔF2-infected lung slice presenting a β-aggregated structure signature. The color is associated to each IR spectrum depending on the score obtained by principal component analysis (from yellow to red). The white dot corresponds to outlier spectrum presenting a β-aggregated structure signature but discarded from the PCA because of the abnormal deformation of the baseline of the spectrum in order to avoid any misinterpretation. Scale bar = 100 µm.

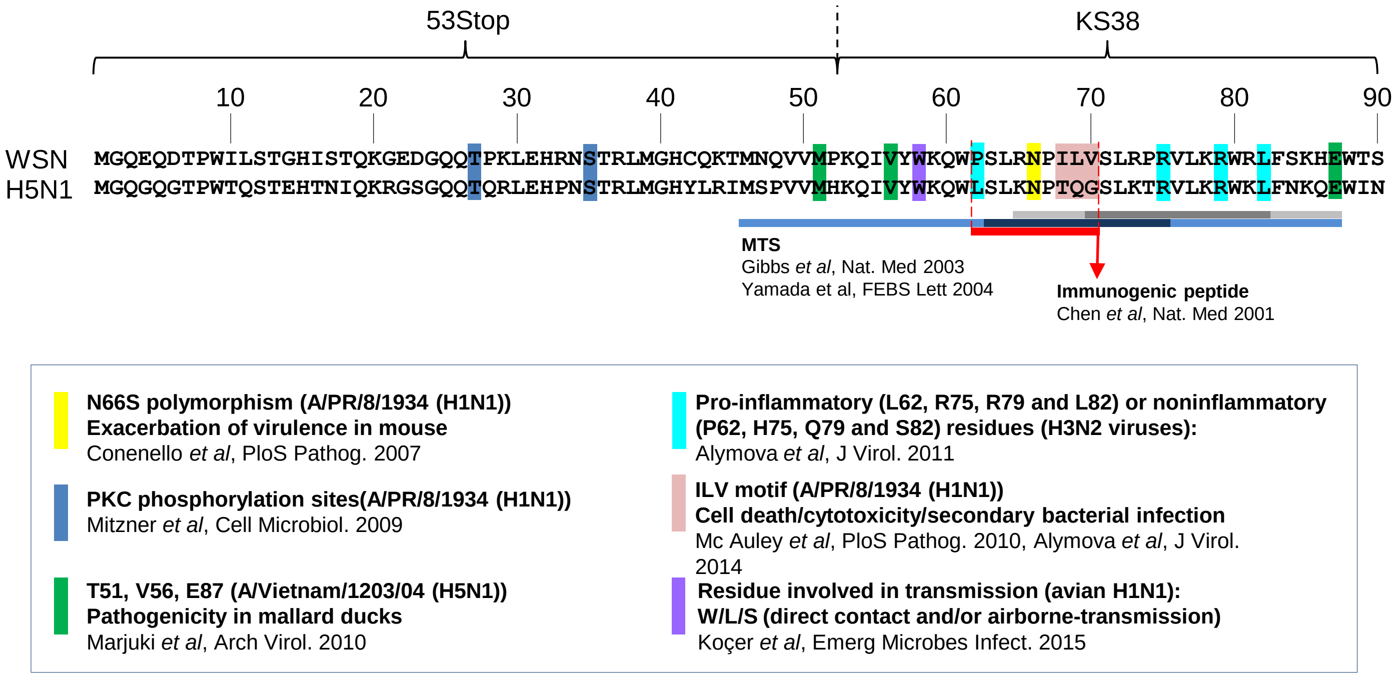

Supplementary figure 3: Alignment of PB1-F2 sequences from A/WSN/1933(H1N1) and A/duck/Niger/2090/2006(H5N1).
Location of key sequences and polymorphisms are indicated with corresponding bibliographic references and the name of the viral strains used in the studies. The immunogenic peptide that allowed to identify PB1-F2 (A/PR/8/1934(H1N1)) for the first time is underlined in red. Peptides predicted to constitute the mitochondrial targeting sequence of PB1-F2 (A/PR/8/19345H1N1)) are underlined in grey and blue (the minimal MTS predicted are underlined with dark colors).

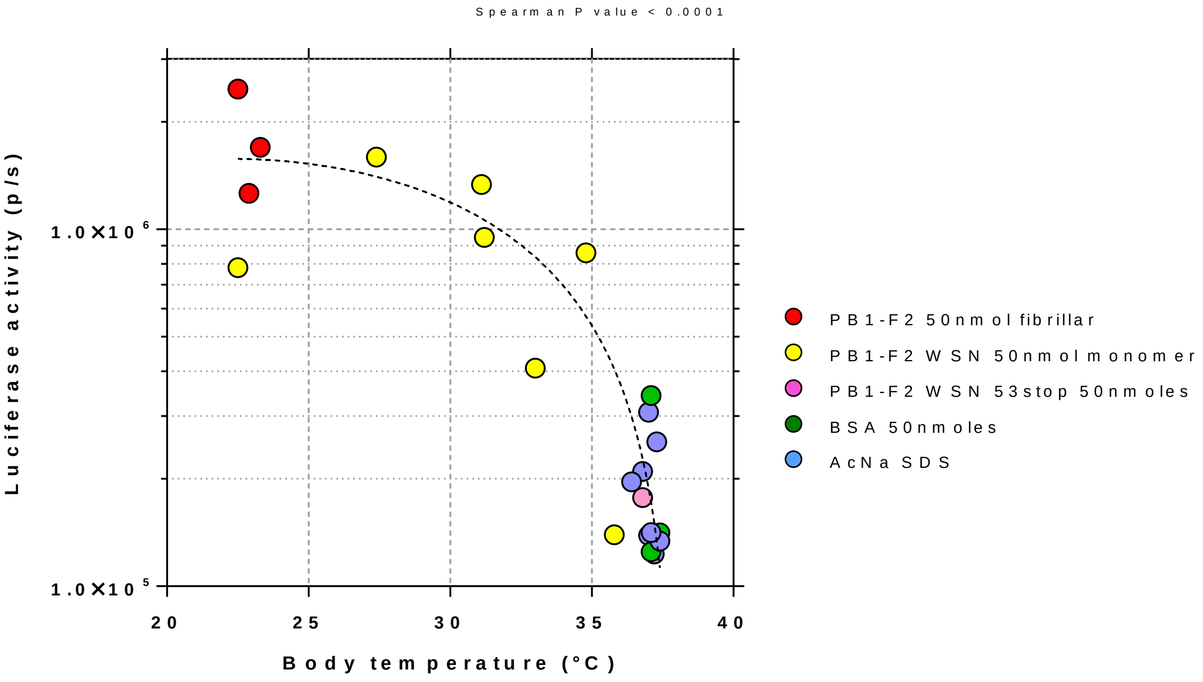

Supplementary figure 4: Correlation of the luciferase activity (*i.e.* NF-kB activity) with body temperature of PB1-F2-instilled mice 18 h post-instillation.

Female NF-kB-Luciferase transgenic BALB/c mice were lightly anesthetized with a mixture of Ketamine and Xylazine (60 mg/kg and 12 mg/kg, respectively) and were intranasally instilled with 50 nmol of PB1-F2 (monomeric, fibrillated or N53Stop truncated forms) in 50 µL of 50 mM sodium acetate pH 5. Control mice were instilled with 50 mM sodium acetate pH 5 alone or complemented with 50 nmol of BSA. 18 h post-instillation, rectal temperatures were recorded and mice were monitored for bioluminescence. Bioluminescence was measured using the IVIS 200 imaging system (PerkinElmer). Mice were anaesthetized and luminescence was measured 5 min after intranasal injection of 50 µL of PBS containing D-luciferin (0.75 mg.kg-1, Sigma). Living Image software (version 4.0, PerkinElmer) was used to measure the luciferase activities. Bioluminescence images were acquired for 1 min with f/stop = 1 and binning = 8. A digital false-color photon emission image of the mouse was generated, and photons were counted within the whole-body area. Photon emission was measured as radiance in $p.s^{-1}.cm^{-2}.sr^{-1}$. A two-tailed nonparametric Spearman test was performed and revealed a high significant correlation between the two data sets: p-value<0,0001.

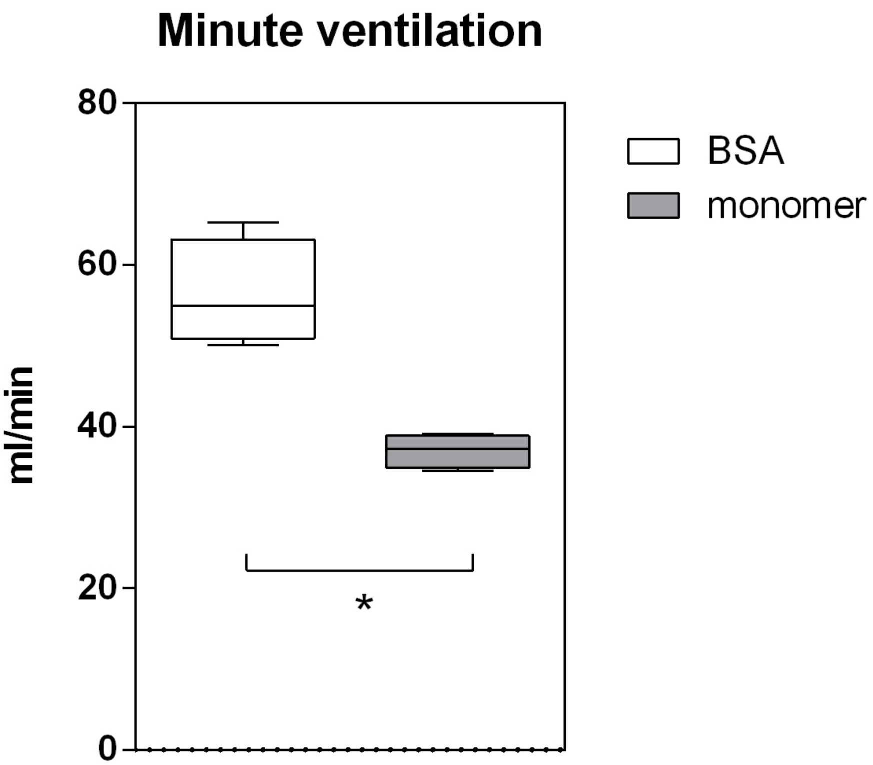

Supplementary figure 5: Minute ventilation of PB1-F2-instilled mice.
Mice were lightly anesthetized with a mixture of Ketamine and Xylazine (60 mg/kg and 12 mg/kg, respectively) and were intranasally instilled with 50 nmol of the monomeric form of PB1-F2 in 50 µL of 50 mM sodium acetate pH 5. Control mice were instilled with 50 mM sodium acetate pH 5 complemented with 50 nmol of BSA. Eighteen h post-instillation, respiratory measurements were acquired by using a whole-body plethysmograph. Vigil mice were placed in chambers that allow measurement of the differential pressure due to breathing of the animal. Following acclimation, respiration rate, tidal volume and minute volume were measured for a 5 min period (* p-value < 0.05).

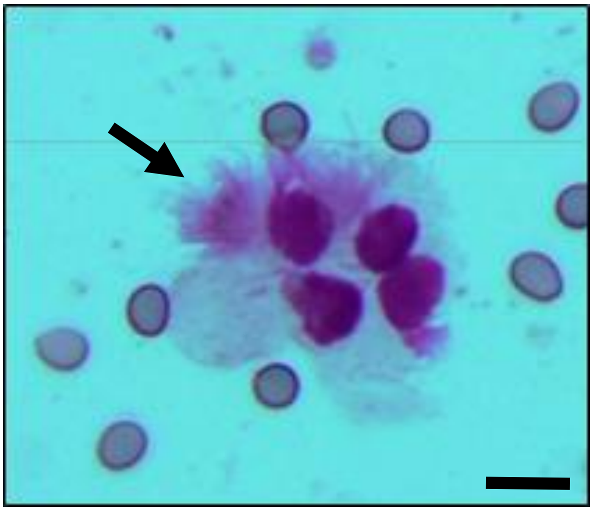

Supplementary Figure 6: Ciliated epithelial cells.
Photomicrographs of ciliated epithelial cells recovered by respiratory lavage from respiratory tract of mock-infected mice. The cells have a cylindrical morphology. Arrows indicate cilia (scale bar = 20 μm).
